# A Gain‐of‐Function Variant in Dopamine D2 Receptor and Progressive Chorea and Dystonia Phenotype

**DOI:** 10.1002/mds.28385

**Published:** 2020-11-16

**Authors:** Marlous C.M. van der Weijden, Dayana Rodriguez‐Contreras, Cathérine C.S. Delnooz, Brooks G. Robinson, Alec F. Condon, Michelle L. Kielhold, Gilles N. Stormezand, Kai Yu Ma, Claudia Dufke, John T. Williams, Kim A. Neve, Marina A.J. Tijssen, Dineke S. Verbeek

**Affiliations:** ^1^ Department of Genetics University Medical Center Groningen Groningen the Netherlands; ^2^ Expertise Center Movement Disorders Groningen University Medical Center Groningen Groningen the Netherlands; ^3^ Department of Behavioral Neuroscience Oregon Health & Science University Portland Oregon USA; ^4^ Department of Neurology Máxima Medical Center Veldhoven the Netherlands; ^5^ Vollum Institute Oregon Health & Science University Portland Oregon USA; ^6^ Department of Nuclear Medicine and Molecular Imaging University Medical Center Groningen Groningen the Netherlands; ^7^ Institute of Medical Genetics and Applied Genomics University Hospital Tuebingen Tuebingen Germany; ^8^ Research Service Virginia Portland Health Care System Portland Oregon USA; ^9^ Department of Neurology University of Groningen, University Medical Center Groningen Groningen the Netherlands

**Keywords:** hyperkinetic movement disorder, chorea, dystonia, dopamine D2 receptor

## Abstract

**Background:**

We describe a 4‐generation Dutch pedigree with a unique dominantly inherited clinical phenotype of a combined progressive chorea and cervical dystonia carrying a novel heterozygous dopamine D2 receptor (*DRD2*) variant.

**Objectives:**

The objective of this study was to identify the genetic cause of the disease and to further investigate the functional consequences of the genetic variant.

**Methods:**

After detailed clinical and neurological examination, whole‐exome sequencing was performed. Because a novel variant in the *DRD2* gene was found as the likely causative gene defect in our pedigree, we sequenced the *DRD2* gene in a cohort of 121 Huntington‐like cases with unknown genetic cause (Germany). Moreover, functional characterization of the *DRD2* variant included arrestin recruitment, G protein activation, and G protein‐mediated inhibition of adenylyl cyclase determined in a cell model, and G protein‐regulated inward‐rectifying potassium channels measured in midbrain slices of mice.

**Result:**

We identified a novel heterozygous variant c.634A > T, p.Ile212Phe in exon 5 of *DRD2* that cosegregated with the clinical phenotype. Screening of the German cohort did not reveal additional putative disease‐causing variants. We demonstrated that the D2_S/L_‐I^212^F receptor exhibited increased agonist potency and constitutive activation of G proteins in human embryonic kidney 239 cells as well as significantly reduced arrestin3 recruitment. We further showed that the D2_S_‐I^212^F receptor exhibited aberrant receptor function in mouse midbrain slices.

**Conclusions:**

Our results support an association between the novel p.Ile212Phe variant in *DRD2*, its modified D2 receptor activity, and the hyperkinetic movement disorder reported in the 4‐generation pedigree. © 2020 The Authors. *Movement Disorders* published by Wiley Periodicals LLC on behalf of International Parkinson and Movement Disorder Society.

Dopamine (DA) regulates diverse physiological functions, including movement, motivation, reward, and learning.^1^ DA acts on its G protein‐coupled DA receptors, D1, D2, D3, D4, and D5,^2^, ^3^ to modulate G protein‐dependent and G protein‐independent signaling cascades. Activation of the former results in dissociation of the G protein αβγ heterotrimer into α and βγ subunits, each of which regulates other signaling proteins. The D2 receptor activates Gα_i/o_ subunits that inhibit adenylyl cyclase thereby decreasing cyclic adenosine monophosphate (cAMP) levels, a key regulator of many intracellular signaling pathways including ion channels and transcription factors.^2^, ^3^ The βγ subunits bind directly to many proteins involved in intracellular signaling, including G protein‐coupled inwardly rectifying potassium channels (GIRKs), which mediate D2 autoreceptor inhibition of DA neuron firing.^4^ One mechanism of G protein‐independent signaling is recruitment of arrestin, which is implicated in both receptor desensitization and activation of G protein‐independent signaling pathways.^5^, ^6^

Dysfunction within dopaminergic pathways is linked to movement disorders including Parkinson's disease and, to a certain extent, Huntington's disease.^7^, ^8^, ^9^ These diseases, and the dopaminergic pathway itself, are also associated with a wide range of psychiatric comorbidities, including depression, anxiety, and hallucinations.^10^ Studies examining the genetic depletion of DA receptors using transgenic mouse models have shown that a loss of D2 receptors results in impaired locomotion that is comparable to the clinical phenotype of Parkinson's disease,^11^, ^12^, ^13^ while upregulation of the D2 receptor in medium spiny projection neurons of the nucleus accumbens in mice results in enhanced locomotion.^14^

Here, we studied a large Dutch family with a dominantly inherited hyperkinetic movement disorder, identified a novel *DRD2* variant that cosegregated with the movement disorder, and excluded the potential existence of a similar genetic defect in a German cohort with Huntington‐like cases without a known genetic cause. The novel D2 receptor variant activates G proteins more efficiently and recruits arrestin3 less effectively than the reference receptor in a cellular model and also exhibits aberrant function in DA neurons in mouse midbrain slices. To the best of our knowledge, this is the first *DRD2* variant with in vitro and ex vivo demonstrated dysfunction to segregate with a movement disorder described in humans.

## 1 Materials and Methods

A full version of the Materials and Methods section can be found in the Supporting Information.

### 1.1 Patient Demographics

A total of 8 family members from this 4‐generation pedigree underwent clinical anamnesis and a standardized neurological examination by 2 neurologists (C.C.S. and M.A.J.) who were blinded to patient and relationship status. Their basic characteristics are described in Table 1. In addition, cognitive function was tested using the Mini‐Mental State Examination (MMSE) and Frontal Assessment Battery (FAB). All methods were performed in accordance with the relevant guidelines and regulations. All patients gave written informed consent. In addition, a cohort with Huntington‐like phenotypes was collected from the Institute of Medical Genetics and Applied Genomics of the University of Tuebingen in Germany. This Huntington's disease–like cohort was composed of sporadic and familial cases with chorea of unknown genetic origin. Chorea was the only symptom in 48 patients, 13 patients also had dystonia, 23 patients had accompanying memory problems, 13 patients also had psychiatric problems,13 patients had ataxia as well, 8 patients had accompanying orofacial dyskinesia, and 3 had accompanying tremor. The study was performed in accordance with the Declaration of Helsinki and approved by the Medical Ethical Committees of the University Medical Center Groningen and the University of Tuebingen.

**TABLE 1:** Patient demographics

Patient			Onset		Symptom				Results of Cosegregation Analysis
Pedigree	Age	Sex	Age	Presenting Symptoms	Chorea	Dystonia	Cognition	Psychiatry	
IV:4	30	Male	14	Chorea of the head, trunk and UE	Head, UE, LE, trunk, vertical eye movement apraxia	Neck, UE, orofacial (tongue deviation to the right)	Cognition intact	No symptoms	59662A > AT,c.634A > T, p.Ile212Phe
IV:5	29	Female	18	Irregular movements of the head, trunk, UE and LE	Head, UE, LE, trunk, vertical eye movement apraxia	Neck	Cognition intact	No symptoms	59662A > AT,c.634A > T, p.Ile212Phe
III:4	64	Female	Childhood	Irregular movements of the head and UE	Head, UE, LE, orofacial dyskinesias of the mouth	Neck, UE, dystonic posturing of the right arm and digits	MMSE intact, FAB intact	Vivid dreams	59662A > AT,c.634A > T, p.Ile212Phe
III:6	62	Female	Childhood	Irregular movements of the head, dystonic posturing of the head	Head, trunk, orofacial	Neck	MMSE intact FAB intact	Agoraphobia	59662A > AT,c.634A > T, p.Ile212Phe
III:8^a^	60	Female	20	Chorea and dystonic posturing of the head	Head, UE, LE, trunk, orofacial, eye movement apraxia	Neck	Memory problems	Generalized anxiety disorder/panic attacks	59662A > AT,c.634A > T, p.Ile212Phe
III:10	59	Female	n.a.	n.a.	No symptoms	No symptoms	MMSE intact FAB intact	Burn‐out	No mutation
III:13	57	Female	n.a.	n.a.	No symptoms	No symptoms	MMSE intact FAB intact	Burn‐out	No mutation
III:14	47	Male	n.a.	n.a.	No symptoms	No symptoms	MMSE intact FAB intact	No symptoms	No mutation

*Note*. Patient demographics of clinical symptoms and age of onset. Information about mutation analysis was performed in 8 family members who gave written informed consent. Roman digits correspond to pedigree of Figure 1.

Abbreviations: UE, upper extremities; LE, lower extremities; n.a., not applicable; MMSE, Mini‐Mental State Examination; FAB, Frontal Assessment Battery.

^a^ Proband.

### 1.2 Genetic Studies

The proband of the family was tested for an in‐house dystonia gene panel (Table S1) and repeat expansions (CAG, CAA, CTG) in *HTT*; Huntington's disease‐like (HDL) genes including *PRNP* (HDL‐1), *JPH3* (HDL‐2), and *TBP* (HDL‐4/ Spinocerebellar ataxia (SCA) type 17); and benign hereditary chorea (*NKX2‐1*) (Table S2). When these tests came back negative, whole‐exome sequencing (WES) was performed using Macrogen (Macrogen Inc.). Data derived from WES were analyzed using Cartagenia software (Agilent Technologies). The p.Ile212Phe variant was confirmed using Sanger sequencing with forward 5'GGACATGAATGGGCTCTTGT3′ and reverse 5′TCCTGGGAATTCCTTTAGCC3′ primers. In the German cohort, sequencing of the *DRD2* coding region was performed using Sanger sequencing (for primer sequences, see Table S3). Mutation analysis was performed using Mutation Surveyor version 5.1.2 (Softgenetics LLC).

### 1.3 Positron Emission Tomography Scan Imaging

In vivo D2 receptor positron emission tomography (PET) imaging was performed using [^11^C]raclopride, a selective DRD_2/3_ antagonist, on individuals III:8, IV:4, and IV:5. Subjects were asked to refrain from smoking for 12 hours and from drinking alcohol for 24 hours and to not eat 4 hours prior to the PET scan. Striatal DA D_2/3_ receptor availability was measured following a 60‐minute dynamic acquisition protocol after a 1‐minute bolus injection of 200 (204–220) MBq of [^11^C]raclopride on a Siemens Biograph mCT system (Siemens Medical Solutions USA, Inc). All images were spatially normalized using PMOD (PMOD Technologies Ltd). Brain regions were defined using the Hammers Atlas. Binding potentials (BPs) were calculated for each patient using a simplified reference tissue model, using the cerebellum as the reference region, and compared with BPs of healthy subjects (healthy, nonsmoking, nonmedicated subjects). Age‐dependent decline of BP values was taken into account as described in Nakajima and colleagues.^15^

### 1.4 Recombinant cDNA Constructs

Descriptions of the plasmids used in this study can be found in the Supporting Information.

### 1.5 Bioluminescence Resonance Energy Transfer Assays

After 48 hours of transfection, human embryonic kidney 239 (HEK293) cells were harvested, washed, and resuspended in phosphate‐buffered saline containing 0.1 mM CaCl_2_ and 0.5 mM MgCl_2_ and plated at ~100,000 cells/well in 96‐well OptiPlates (PerkinElmer Life Sciences). Emission of the donor (460 μm) and acceptor (535 μm) was measured at several timepoints after adding quinpirole followed by the luciferase substrate coelenterazine *h* at room temperature, and bioluminescence resonance energy transfer (BRET) ratios were calculated as previously described.^16^, ^17^

### 1.6 Data Analysis

Concentration‐response curves and radioligand saturation binding curves were analyzed by nonlinear regression using Prism 7 or 8 (GraphPad Software Inc.). Statistical significance between 2 groups was determined using the Student's *t*‐test. For comparisons with more than 2 groups, we used analysis of variance followed by the Tukey's multiple comparisons test. For [^3^H]spiperone binding, the geometric mean (mean of logKd) was calculated and used for statistical comparison.

## 2 Results

### 2.1 Identification of a Family with Unique Hyperkinetic Movement Disorder

Patient demographics are presented in Table 1. The proband (III:8; Fig. 1A) visited our outpatient clinic at age 60 years. Her history revealed dance‐like irregular movements and abnormal posturing of the head that started at age 20 years, progressed over time, and extended to orofacial involvement (Video S1). In addition, the subject reported that, with increasing age, she developed memory problems and generalized anxiety with panic attacks. Generalized anxiety was treated with sertraline, which gave no perceptible relief of the irregular movements. Neurological examination showed choreatic movements of the orofacial region and all extremities, dystonic posturing of the head, and eye movement apraxia. Cognitive function (MMSE and FAB) was normal. The proband's older sister by 2 years (III:6; Fig. 1A) was noted to have abnormal posturing of the head and wringing movements of the hands in early childhood. Her symptoms progressed over time, and during stressful situations she developed involuntary movements of the eyebrows and upper lip. Starting at age 30, she suffered from agoraphobia, which was treated with paroxetine but did not lead to reported improvement of the irregular movements. At age 62, neurological examination revealed a phenotype closely resembling the proband's (III:8), with choreatic movements of the trunk, head, and orofacial region and dystonic posturing of the head. Her MMSE and FAB were normal.

**FIG. 1:**
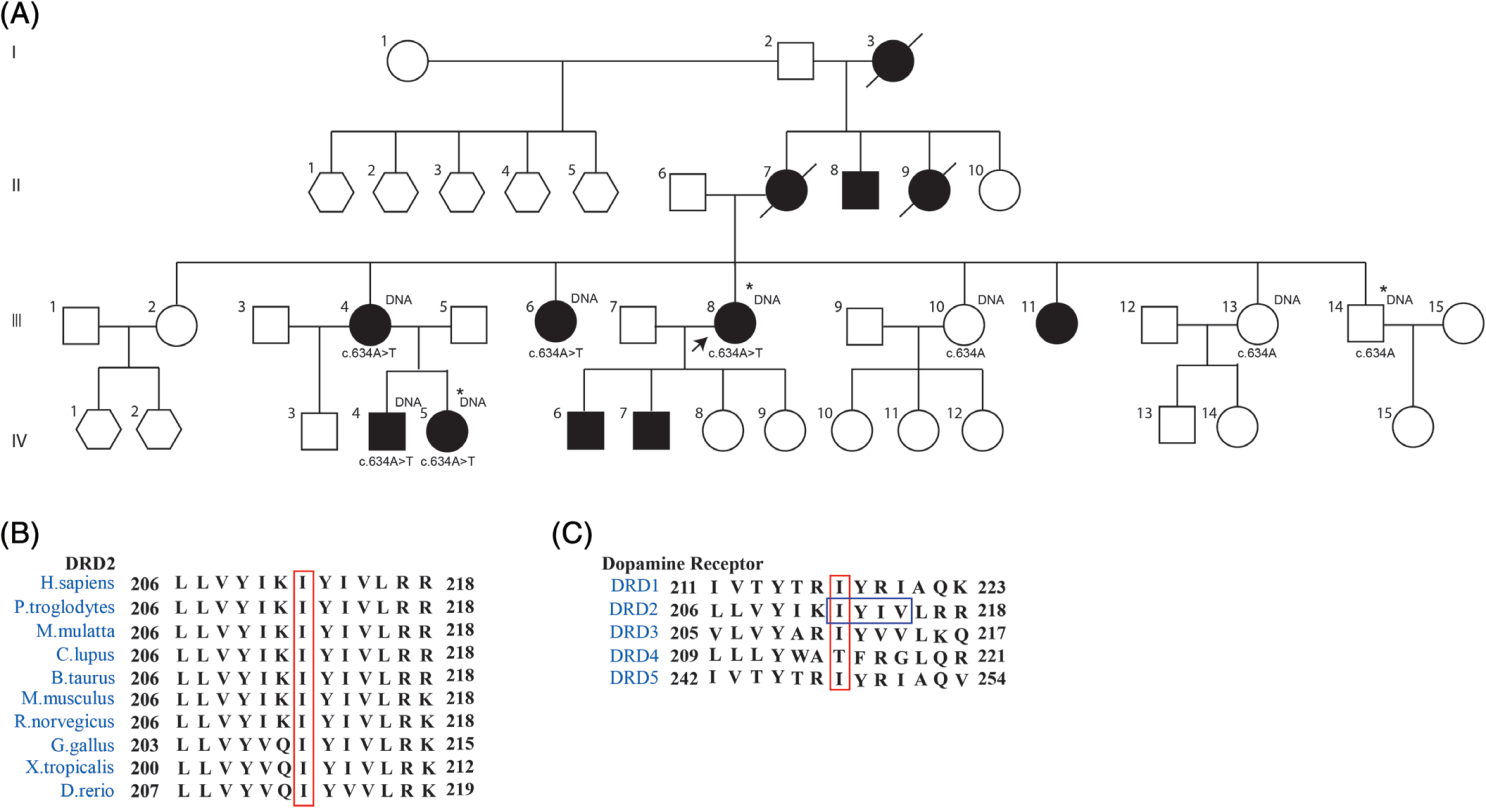
Four‐generation pedigree and amino acid sequence homology. (**A**) Four‐generation pedigree carrying the missense variant c.634A > T, p.Ile212Phe in *DRD2*. Genetic analysis was performed in the individuals indicated by “DNA.” Proband is indicated by an arrow. Individuals marked by an asterisk underwent whole‐exome sequencing. The variant (c.634A > T) was identified in 5 affected subjects and was absent in 3 unaffected subjects. Male = square, female = circle, sex unknown = hexagon. Filled symbols = affected. Open symbols = unaffected. Sequence alignments showing the amino acid sequence identity of the affected amino acids in (**B**) multiple species orthologs of DRD2 and (**C**) among the human dopamine receptor family. The mutated amino acid isoleucine at position 212 is indicated by the vertical box. The region of arrestin‐binding (IYIV) in *DRD2* is indicated by the horizontal box. [Color figure can be viewed at wileyonlinelibrary.com]

The eldest affected sister (III:4) developed progressive irregular movements of the head, trunk, arms, and hands starting in early childhood, followed by dystonic posturing of the neck and dyskinesia of the mouth. At age 64 years, she exhibited orofacial, head, truncal, and limb choreatic movements. She also developed dystonic posturing of the right arm, right shoulder, and fingers. Her MMSE and FAB were normal.

Affected male IV:4 (Fig. 1A, Table 1) started exhibiting irregular choreatic movements of the head, trunk, and upper extremities at age 14 years. He later developed abnormal posturing of the neck and upper extremities. Upon examination at age 30 years, he had choreatic movements of the head, trunk, and upper extremities and eye movement apraxia. Dystonic posturing of the head, left shoulder, and both arms was also present. His younger sister (IV:5; Fig. 1A, Table 1) developed similar symptoms at age 18 years and also followed a similar course. At examination at age 29 years, she showed mild choreatic movements of the head, trunk, and all extremities together with mild dystonia of the neck and right shoulder. No cognitive or psychiatric symptoms were detected in IV:4 and IV:5.

### 2.2 Putative Pathogenic Novel *DRD2* Variant Segregates with the Clinical Phenotype within the Family

To identify the genetic cause underlying the progressive chorea and dystonia phenotype within this family, and because proband III:8 tested negative for mutations in known genes present in an in‐house gene panel (design 2017, Table S1) and was negative for repeat expansions in the *HTT*, HDL genes *JPH3* (HDL‐2) and *TBP* [HDL‐4/spinocerebellar ataxia (SCA17)], and benign hereditary chorea (*NKX2‐1*) (Table S2), WES was performed in affected individuals III:8 and IV:5 and unaffected family member III:14. We then generated an overview of all heterozygous variants that were shared between the affected cases but not present in the unaffected family member. After removing all variants annotated as benign in our in‐house genetic pipeline and all variants present in the GnomAD browser (assessed December 2018), we identified 4 missense variants. Two had a relatively high minor allele frequency and were therefore excluded as potential candidates, and 1 variant turned out to be a false positive and was therefore excluded (Table S4). The fourth variant was a novel heterozygous variant in exon 5 of *DRD2* [126450]: c.634A > T, p.Ile212Phe. Segregation of this variant with the clinical phenotype was confirmed in a total of 5 clinically affected and 3 clinically unaffected family members (Fig. 1A). The pathogenicity of this variant was assessed by the in silico prediction programs Sorting Intolerant From Tolerant (SIFT), Polyphen2, and MutationTaster and the tool Combined Annotation Dependant Depletion (CADD) (Table S5), which all predicted the variant to be damaging. The putative pathogenicity of the novel *DRD2* variant is further substantiated by the fact that the exchange of amino acids in the 211–213 region seems quite intolerant, as no missense variants are reported at these positions in GnomAD (assessed January 2020). Moreover, the isoleucine at this position is highly conserved among orthologous genes of the D2 receptor (Fig. 1B) and among human DA receptor subtypes (Fig. 1C), with the exception of the D4 receptor. The variant results in a change of the strongly hydrophobic amino acid isoleucine at position 212 into a hydrophobic and aromatic phenylalanine that is predicted to mildly alter the secondary structure of this intracellular extension of the fifth transmembrane domain (TM5) (Grantham score 21).

### 2.3 Variants in *DRD2* May Only Rarely Associate with Huntington‐Like Phenotypes

To assess whether this variant was present in other populations with Huntington‐like phenotypes, we screened a cohort of 121 DNA samples collected by the Institute of Medical Genetics and Applied Genomics of the University of Tuebingen, Germany, for rare variants throughout the coding region of *DRD2* (see Materials and Methods). We identified several relatively common variants in multiple cases and 1 rare variant, c.62517C > T, p.Pro347Ser in exon 7 of *DRD2*, that was predicted in silico to be benign (Table S6).

### 2.4 Cerebral D2 Receptor Levels Are Not Altered in Patients

To assess the impact of variant c.634A > T, p.Ile212Phe on cerebral DRD2 expression levels, we performed D2 receptor PET imaging using [^11^C]raclopride, a selective DRD_2/3_ antagonist, on individuals III:8, IV:4, and IV:5. When an age‐dependent decline was taken into account as described in Nakajima and colleagues,^15^ the BPs of the caudate nucleus of patients were within normal range (Table S7). When age‐dependent declines in BPs of the putamen were considered, patient values were close to the lower limit of the normal range but were still within normal range.

### 2.5 The p.Ile212Phe Variant Impairs D2 Receptor Recruitment of Arrestin3

**FIG. 2:**
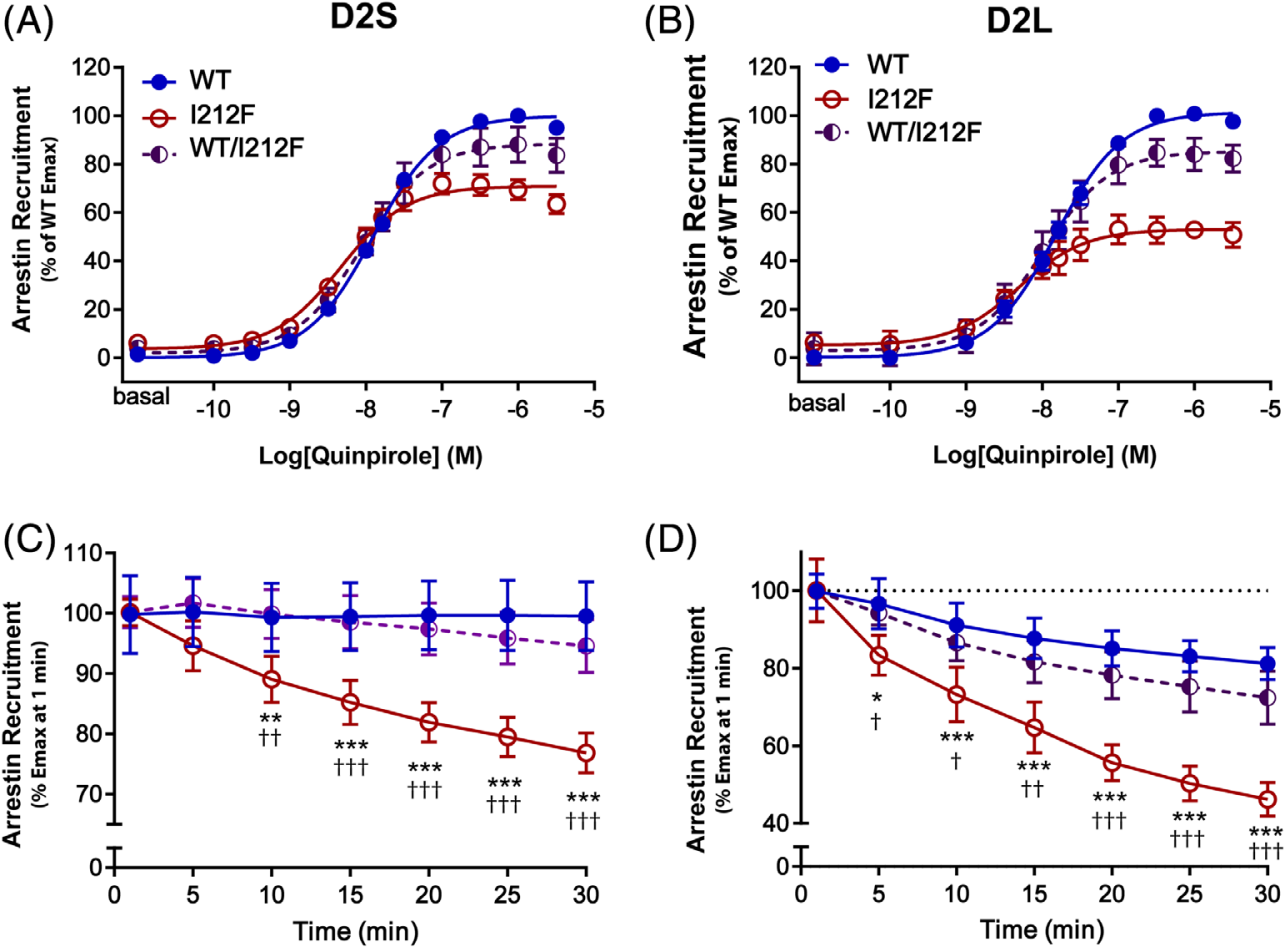
Arrestin recruitment in response to quinpirole. (**A,B**) Dose‐response curves for arrestin recruitment mediated by D2‐WT, D2‐I^212^F, and D2‐WT/I^212^F in response to a 20‐minute stimulation with quinpirole. Results are expressed as a percentage of maximum arrestin recruitment by D2‐WT. (**A**) Data for D2_S_. (**B**) Data for D2_L_. (**C,D**) Time course of maximal recruitment of arrestin for each condition. Basal response was subtracted from the maximal quinpirole stimulation response for each indicated time. Maximal recruitment was normalized to its initial response (1‐minute response). There was a significant interaction between genotype and time for maximal recruitment (2‐way analysis of variance: *F*_12,63_ = 3.628, *P* = 0.0004 for D2_S_, and *F*_12,42_ = 4.304, *P* = 0.0002 for D2_L_). Values plotted are mean ± SD of 4 (**A,C**) and 3 (**B,D**) independent experiments performed in quadruplicate. *P < 0.05, **P < 0.01, and ***P < 0.001 compared to D2‐WT and †*P* < 0.05, ††*P* < 0.01, and †††*P* < 0.001 compared to D2‐WT/I^212^F, Tukey's multiple comparisons test. Emax, maximum response; WT, wild type. [Color figure can be viewed at wileyonlinelibrary.com]

To investigate the effect of the sequence polymorphism on D2 receptor function, we investigated the ability of the mutant D2 receptor to recruit arrestin3. We hypothesized that the arrestin3 recruitment may be affected, as the p.Ile212Phe variant is located in a 4 amino acid motif (212–215), which was previously linked to arrestin3 recruitment to the D2 receptor.^17^, ^18^ Arrestin3 recruitment is required for terminating G protein‐coupled receptor signaling, facilitating receptor internalization, and engaging noncanonical, G protein‐independent signaling pathways.^19^ Because the D2 receptor has alternatively spliced long and short isoforms that differ in the third cytoplasmic loop, which is involved in binding arrestin and G proteins, we tested the consequence of the p.Ile212Phe variant in both. The D2 receptor agonist quinpirole produced a concentration‐dependent increase in arrestin3 recruitment for both wild type (WT) and I^212^F D2 receptors, but maximal recruitment of arrestin3 by D2_S_‐I^212^F and D2_L_‐I^212^F was 68% ± 1% and 48% ± 2%, respectively, of the maximal recruitment by D2_S/L_‐WT (Fig. 2A,B; Table S8). There was also a significant interaction between genotype and the time course of maximal arrestin3 recruitment (Fig. 2 legend), with the BRET signal decreasing more rapidly for D2_S/L_‐I^212^F than for D2_S/L_‐WT (Fig. 2C,D). In addition, the potency of quinpirole was modestly but significantly enhanced for D2_S/L_‐I^212^F compared with D2_S/L_‐WT (Table S8). The membrane expression of D2_S_‐I^212^F and D2_L_‐I^212^F receptors was 50% and 38% of D2_S/L_‐WT expression, respectively (Table S9), but did not explain the lowered ability to recruit arrestin3 by D2‐I^212^F, as a similar impairment of arrestin3 recruitment was observed in an independent experiment where a doubled amount of D2_L_‐I^212^F DNA was transfected (data not shown). Coexpression of D2‐WT and D2‐I^212^F (D2_S_‐WT/I^212^F and D2_L_‐WT/I^212^F) in cells resulted in arrestin3 recruitment that was more similar to D2‐WT than to D2‐I^212^F (Fig. 2A‐D; Table S8).

### 2.6 D2‐I^212^F Receptors Exhibit Increased Basal G Protein Activation and Enhanced Agonist Potency

**FIG. 3:**
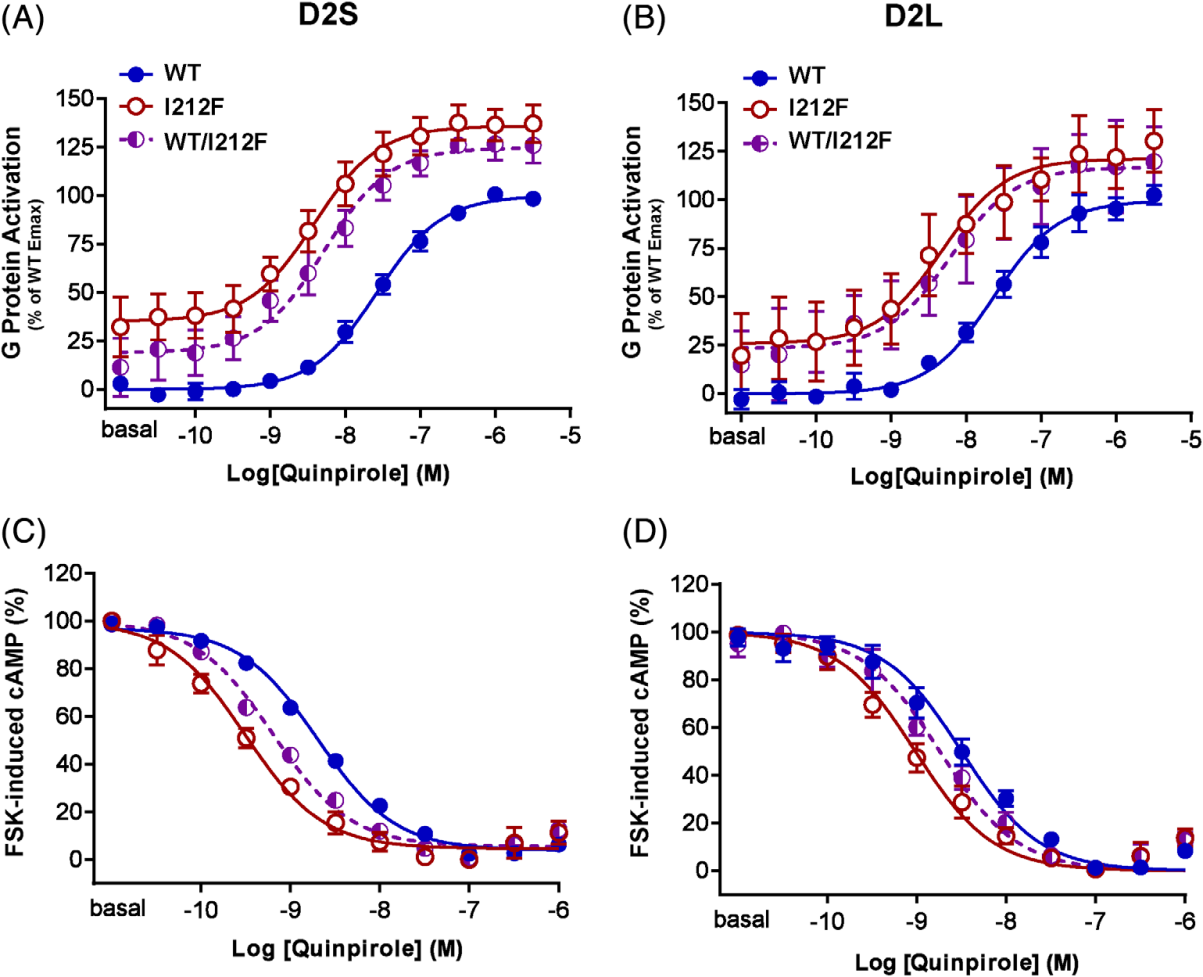
Protein activation in response to quinpirole. (**A,B**) Dose‐response curves for Gα_i1_ protein activation mediated by D2‐WT, D2‐I^212^F, and D2‐WT/I^212^F in response to a 10‐minute stimulation with quinpirole. Results are expressed as a percentage of maximum G protein–activation by D2‐WT. Values plotted represent mean ± SD of 4 independent experiments performed in quadruplicate. (**C,D**) Dose‐response curves for the inhibition of forskolin‐stimulated cAMP accumulation mediated by D2‐WT, D2‐I^212^F, and D2‐WT/I^212^F in response to incubation with quinpirole for 10 minutes in the presence of 10 μM forskolin. Results are expressed as a percentage of maximum cAMP accumulation for each condition. Values plotted are mean ± SD of 4 (**C**) and 6 (**D**) independent experiments performed in triplicate. Left and right panels depict data for D2_S_ and D2_L_, respectively. cAMP, cyclic adenosine monophosphate; Emax, maximum response; FSK, forskolin; WT, wild type. [Color figure can be viewed at wileyonlinelibrary.com]

To investigate how the p.Ile212Phe variant in *DRD2* could affect the Gα_i/o_ protein–mediated signaling pathway, we measured the ability of D2‐I^212^F to activate Gα_i1_ protein and to inhibit forskolin‐stimulated activation of adenylyl cyclase. The potency of quinpirole for activation of Gα_i1_ was significantly increased for D2‐I^212^F (both D2_S_ and D2_L_) relative to D2‐WT, reflected in the leftward shift in the dose‐response curve (Fig. 3A,B; Table S8). The shift in potency was also seen in cells coexpressing D2_S_‐WT/I^212^F and D2_L_‐WT/I^212^F. Cells expressing D2‐I^212^F also exhibited increased basal G protein activation (Fig. 3A,B; mean ± SEM: −0.01% ± 0.01% of maximal stimulation for D2_S_‐WT vs. 35% ± 6% for D2_S_‐I^212^F, *P* < 0.001, n = 4, and 0.01% ± 0.02% for D2_L_‐WT vs. 26 ± 10% for D2_L_‐I^212^F, *P* < 0.05, n = 4). G protein‐coupled receptors exhibit differing degrees of constitutive activity (ie, signaling in the absence of agonist), and many mutations increase constitutive activity.^20^ Upon correction for baseline activity, maximal activation of Gα_i1_ did not differ significantly among the different genotypes (Table S8). The increased agonist potency and constitutive activity occurred despite significantly lower membrane expression of D2‐I^212^F compared with D2‐WT (Table S9).

We then investigated cAMP accumulation in cells expressing D2_S/L_‐WT, D2_S/L_‐I^212^F or D2_S/L_‐WT/I^212^F and cAMP sensor using YFP‐Epac‐Rluc (CAMYEL), a BRET‐based cAMP sensor.^17^ D2_S/L_‐I^212^F showed increased quinpirole potency for inhibiting forskolin‐induced cAMP compared with D2_S/L_‐WT, reflected by left‐shifted concentration‐response curves (Fig. 3C,D; Table S8). No significant difference was observed between D2‐WT and D2‐I^212^F in maximal inhibition of cAMP accumulation (Table S8). In these experiments, D2_S/L_‐I^212^F was again expressed less abundantly at the plasma membrane compared with D2_S/L_‐WT (Table S9). D2_S/L_‐WT/I^212^F significantly differed in quinpirole potency compared with both D2_S/L_‐WT and D2_S/L_‐I^212^F (Table S8).

### 2.7 Altered D2‐I^212^F Receptor‐GIRK Currents and Inhibitory Postsynaptic Currents in Mouse Midbrain Slices

**FIG. 4:**
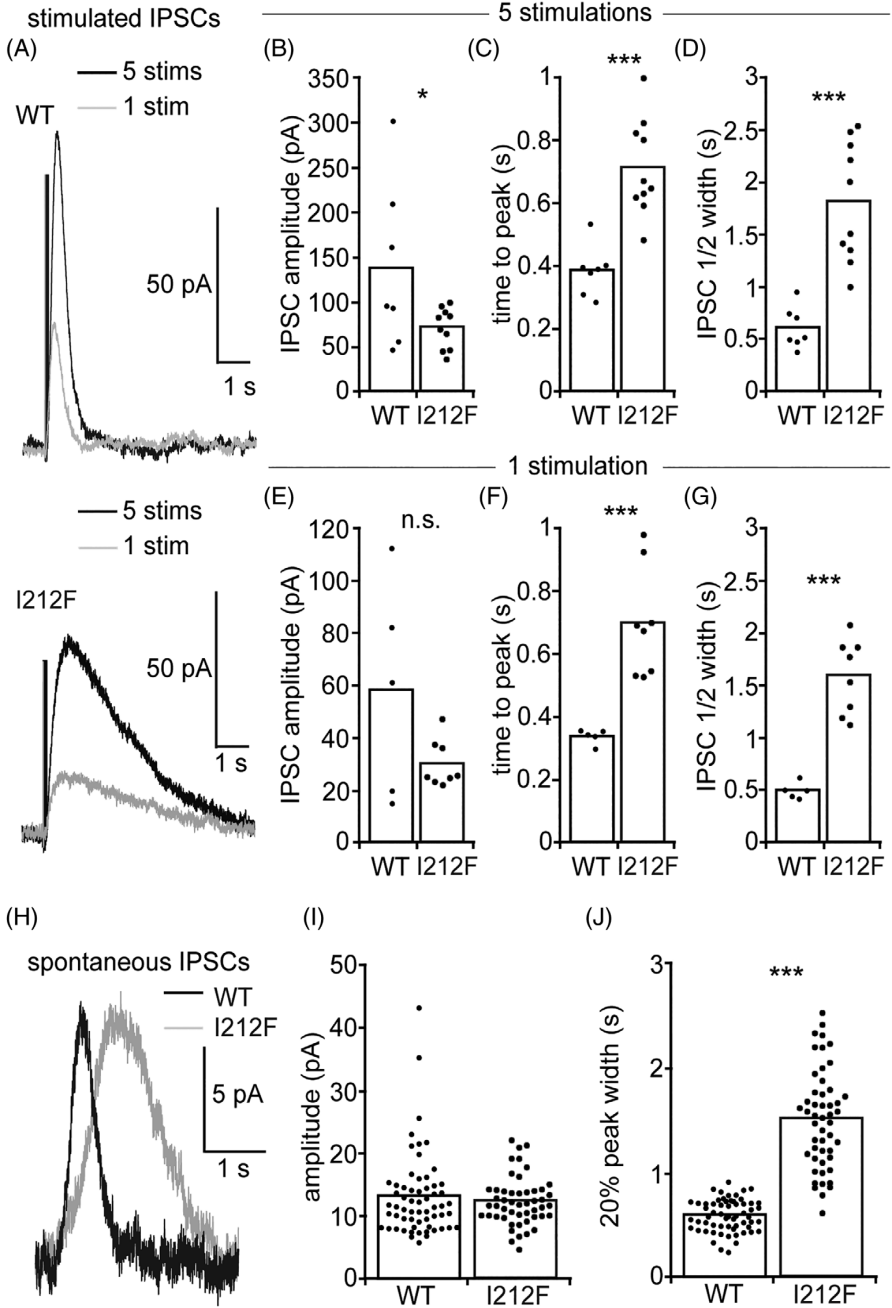
D2_S_ receptor‐GIRK IPSCs in dopamine neurons. (**A**) Example recordings of electrically stimulated IPSCs from neurons expressing D2_S_‐WT (5 stimuli at 40 Hz [black] and 1 stimulus [gray]) and D2_S_‐I^212^F (5 stimuli at 40 Hz [black] and 1 stimulus [gray]). When 5 stimuli were used to elicit IPSCs, neurons expressing D2_S_‐WT had (**B**) larger amplitude IPSCs (Student's*t*‐test; *t* = 2.2141, *P* = 0.043), (**C**) faster time to peak (Student's *t‐*test; 5 stimulations, *t* = 5.1519, *P* < 0.001), and (**D**) shorter half‐width compared to IPSCs in neurons expressing D2_S_‐I^212^F (N = 7 cells from 3 animals for D2_S_‐WT, N = 10 cells from 3 animals for D2_S_‐I^212^F; Student's t‐test; for 5 stimulations, *t* = 5.3117, *P* < 0.001). When 1 stimulus was used to elicit IPSCs, (**E**) neurons expressing D2_S_‐WT and D2_S_‐I^212^F had IPSCs that did not differ significantly in amplitude (Student's *t‐*test; *t* = 1.8838, *P* = 0.086), but (**F**) the IPSC time to peak (Student's *t*‐test; 1 stimulation, *t* = 4.5137, *P* < 0.001) and (**G**) half‐widths were significantly shorter in neurons expressing D2_S_‐WT compared with D2_S_‐I^212^F (N = 5 cells from 2 animals for D2_S_‐WT, N = 8 cells from 3 animals for D2_S_‐I^212^F; Student's *t*‐test; for 1 stimulation, *t* = 6.6569, *P* < 0.001). (**H**) Averages of spontaneous D2 receptor IPSCs from DA neurons expressing D2‐WT (black) or D2‐I^212^F (gray). (**I**) The average spontaneous IPSC amplitudes did not differ between neurons expressing D2_S_‐WT and D2_S_‐I^212^F (N = 61 events from 3 animals for D2_S_‐WT, N = 50 events from 5 animals for D2_S_‐I^212^F; Student's *t*‐test; *t* = 0.72164, *P* = 0.4721). (J) The average spontaneous IPSC width at 20% of peak was significantly longer in DA neurons expressing D2_S_‐I^212^F compared with those expressing D2_S_‐WT (N = 61 events from 3 animals for D2_S_‐WT, N = 50 events from 5 animals for D2_S_‐I^212^F; Student's *t‐*test; *t* = 14.341, *P* < 0.0001). *P < 0.05; **P < 0.01; ***P < 0.001 compared with D2‐WT. IPSCs, inhibitory postsynaptic currents; n.s., not significant; stims, stimulations; WT, wild type.

D2 autoreceptors on DA neurons in the midbrain produce G protein‐mediated activation of GIRK currents that hyperpolarize the neurons and thus decrease cell firing and DA release.^4^, ^21^ To investigate the effect of the p.Ile212Phe variant on D2 receptor activity in a more physiological environment, we assessed D2 receptor regulation of GIRKs in neurons.^22^ We used an adeno‐associated virus (AAV) vector to express D2_S_‐WT or D2_S_‐I^212^F in DA neurons present in midbrain slices of mice in which the D2 receptor has been genetically deleted (Supporting Information Materials and Methods). Whole‐cell voltage‐clamp recordings were performed in green fluorescent protein(GFP)‐marked neurons expressing either D2_S_‐WT or D2_S_‐I^212^F. D2 receptor‐GIRK currents in response to bath‐applied DA or quinpirole were significantly smaller in DA neurons expressing D2_S_‐I^212^F compared with those expressing D2_S_‐WT (Fig. S1B,E). During continued agonist application, the D2‐GIRK currents declined equally in amplitude for D2_S_‐WT and D2_S_‐I^212^F (Fig. S1C). We then electrically stimulated the slice to produce D2 receptor‐GIRK inhibitory postsynaptic currents (IPSCs) in response to somatodendritic DA release. In neurons expressing D2_S_‐I^212^F, the IPSCs peaked later (for both 1 and 5 pulses at 40 Hz; Fig. 4C,F) and had significantly longer half‐widths (Fig. 4D,G) compared with D2_S_‐WT. In addition, 5‐pulse IPSCs were significantly larger for D2_S_‐WT compared with D2_S_‐I^212^F (Fig. 4B), although not for 1 pulse IPSCs (Fig. 4E). While recording, spontaneous D2 receptor‐GIRK IPSCs^23^ were detected in DA neurons expressing either D2_S_‐WT or D2_S_‐I^212^F (Fig. 4H). The amplitudes of the spontaneous IPSCs did not differ between DA neurons expressing D2_S_‐WT or D2_S_‐I^212^F (Fig. 4I), but their width was significantly greater in neurons expressing D2_S_‐I^212^F (Fig. 4J).

## 3 Discussion

We here report the identification of a gain‐of‐function variant in *DRD2* that cosegregates with a mixed phenotype of chorea and dystonia in a large Dutch family. The putative pathogenic effect of the novel c.634A > T, p.Ile212Phe variant in *DRD2* was supported by in silico prediction models. Screening of a German cohort of Huntington‐like cases for *DRD2* variants suggests that variants in *DRD2* may only rarely associate with Huntington‐like phenotypes. We further provide substantial functional evidence using in vitro and ex vivo models that the clinical phenotype may be the result of a constitutively active D2 receptor leading to over‐stimulation of G protein‐dependent signaling derived from a combination of increased signaling efficiency of the G protein‐dependent signaling itself and reduced activation of arrestin.

The importance of amino acid isoleucine at position 212 for D2 receptor functioning is substantiated by prior work reporting that changing the amino acids 212–215 into alanines results in a D2 receptor that lacks arrestin3 recruitment upon agonist stimulation, whereas mutating the amino acids 213–215 (D2‐A3) or 214–215 (D2‐A2) into alanines only partially decreases agonist‐induced arrestin3 recruitment.^17^, ^18^ The reason that D2‐I^212^F is less expressed at the plasma membrane is not yet known, however this was not a direct cause for the reduced arrestin3 recruitment, as experiments with double the amount of D2_L_‐I^212^F DNA showed a similar impairment of arrestin3 recruitment upon agonist stimulation. Our results indicate that isoleucine at position 212 is key in agonist‐dependent arrestin3 recruitment and binding by the D2 receptor, and when changed into a phenylalanine would lead to aberrant receptor functioning.

The role of arrestin3 in movement regulation is further substantiated in animal models of Parkinson's disease where arrestin3 knockout reduces the beneficial locomotor effect of levodopa and enhances dyskinesias, including tongue protrusions and continuous rapid limb movements, whereas overexpression of arrestin3 reduces dyskinesias while maintaining beneficial locomotor effects of levodopa.^24^ These dyskinesias are equivalent to abnormal orolingual and limb movements in humans and similar to the phenotype seen in the family we are reporting. In addition, expression of a mutant D2 receptor that recruits arrestin3, but has little ability to activate G proteins (D2R‐arrestin‐biased mutant, D2R‐ARB), reverses the reduced locomotion phenotype in D2 receptor knockout mice, and overexpression of D2R‐ARB significantly enhances locomotion compared with control mice.^25^

In our report, 2 of 5 affected family members were diagnosed with an anxiety disorder at a later age. This was not present in the unaffected family members of similar age. *DRD2* has been linked to psychiatric problems in myoclonus dystonia.^26^, ^27^ However, the underlying mechanism is unknown. Within our family constitutive activation of the G protein‐dependent pathway may give a potential explanation for the psychiatric symptoms of generalized anxiety and panic attacks as the current main treatment for psychiatric diseases are DRD2 antagonists. Moreover, mice treated with D2 receptor antagonists show reduced anxiety measured by elevated plus maze test scores.^28^, ^29^ This effect was also observed in patients with psychotic disorders, where DA antagonists reduce social anxiety.^30^ Together, these lines of evidence suggest that arrestin3‐mediated D2 receptor signaling regulates locomotion, while dyskinesia is a consequence of G protein‐activation.

Our current results of G protein activation support the literature findings. Together, the G protein‐activation and cAMP data strongly suggest enhanced efficiency of coupling of D2_S/L_‐I^212^F to G proteins compared with D2_S/L_‐WT. Thus, the D2‐I^212^F receptor may be biased towards G protein‐mediated signaling, in part reflecting increased D2 receptor constitutive activity.

To further evaluate D2 receptor activation, we assessed D2 receptor regulation of GIRKs in DA neurons. The data derived from the midbrain slices of mice expressing D2‐WT or D2‐I^212^F support the notion of aberrant G protein‐mediated signaling by D2_S_‐I^212^F. We hypothesize that the reduced peak current amplitude under some conditions in DA neurons expressing D2‐I^212^F may reflect low receptor expression as seen in HEK293 cells. The time to peak and IPSC half‐width were consistent between 1‐pulse or 5‐pulse stimulations for D2_S_‐I^212^F, indicating that the prolonged IPSC mediated by D2_S_‐I^212^F reflects a change intrinsic to the receptor‐GIRK interaction rather than altered DA release or uptake. The increased peak width seen in IPSCs mediated by D2‐I^212^F is consistent with enhanced activation of G proteins by D2‐I^212^F. The slow onset and decay of the response to D2‐I^212^F may indicate that recruitment of arrestin is responsible for the rapid on and off rates of response to D2‐WT.

Unfortunately, no additional Huntington's disease–like cohorts were available for screening besides the cohort from Germany. Future studies are needed to confirm whether mutations in *DRD2* are a more common cause of chorea. When a patient with an early onset autosomal dominant chorea‐dystonia phenotype presents at any outpatient clinic, we may recommend to test first for repeat expansions in early‐onset chorea genes, including *HTT*, HDL genes, and benign hereditary chorea genes, followed by an up‐to‐date dystonia gene panel and subsequently Human Phenotype Ontology (HPO)‐labelled dystonia and chorea genes.

In short, the D2 receptor is well established in regulation of movement, and overstimulation of G protein‐dependent signaling by the mutant D2 receptor together with reduced arrestin recruitment and activation may be the cause of the hyperkinetic movement disorder described in this study. Our findings may have important therapeutic implications because a biased D2 receptor ligand that decreases G protein‐mediated signaling, while sparing arrestin‐mediated signaling, might be an effective treatment in hyperkinetic movement disorders.

## Authors Roles

(1) Research Project: A. Conception, B. Organization, C. Execution; (2) Experimental Procedures and Statistical Analysis: A. Design, B. Execution, C. Review and Critique; (3) Manuscript: A. Writing of the First Draft, B. Review and Critique.

M.V.W.: 1A, 1B, 1C, 2A, 2B, 2C, 3A, 3B

D.R.C.: 1C, 2A, 2B, 2C, 3A, 3B

C.C.S.: 1C, 2A, 2B, 2C, 3B

B.G.R.: 1C, 2A, 2B, 2C, 3B

A.F.C.: 1C, 2A, 2B, 2C, 3B

M.L.K.: 1C, 2A, 2B, 2C, 3B

G.N.S.: 1C, 2A, 2B, 2C, 3B

K.Y.M.: 1C, 2A, 2B, 2C, 3B

C.D.: 1C, 2A, 2B, 2C

J.T.W.: 1C, 2A, 2B, 2C, 3B

K.A.N.: 1B, 1C, 2A, 2B, 2C, 3A, 3B

M.A.J.: 1A, 1B, 1C, 2A, 2B, 2C, 3A, 3B

D.S.V.: 1A, 1B, 1C, 2A, 2B, 2C, 3A, 3B

## 4 Financial disclosures

MD‐PhD scholarship from the University Medical Center Groningen (M.V.W.); Rosalind Franklin Fellowship from the University of Groningen (D.S.V.); K99DA044287 (B.G.R.) and F31DA047007 (A.F.C.) training awards from the U.S. Public Health Service; and grants from the Netherlands Organization for Health Research and Development ZonMW Topsubsidie (91218013) (M.A.J.), the European Fund for Regional Development from the European Union (01492947) and the province of Friesland (M.A.J.), the Dystonia Medical Research Foundation (M.A.J.), the Stichting Wetenschapsfonds Dystonie Vereniging (M.A.J.), the Fonds Psychische Gezondheid (M.A.J.), the Phelps Stichting (M.A.J.), an unrestricted grant from Actelion (M.A.J.); and a Merit Review Award BX003279 from the US Department of Veterans Affairs, Veterans Health Administration, Office of Research and Development, Biomedical Laboratory Research and Development (K.A.N.).

## Supporting information


**Appendix S1** Supporting Information.

**Video S1** Video of the index patient. The first part of the video shows choreatic movements of the orofacial region and upper extremities as well as dystonic posturing of the head. In the second part, the patient is asked to stretch her arm and count backwards. Here the choreatic symptoms are shown in the trunk and lower extremities. In the last part, the patient is asked to walk. Here, dystonic posturing and tremor of the head is visible as well as chorea of the upper extremities.
