# Supplementary material for: A Gain‐of‐Function Variant in Dopamine D2 Receptor and Progressive Chorea and Dystonia Phenotype
Source: Mov Disord. 2020 Nov 16;36(3):729–39. doi: 10.1002/mds.28385 (PMC8049080; doi:10.1002/mds.28385)
Supplement: Supplementary file 1 — Appendix S1 Supporting Information. [file MDS-36-729-s002.docx]

**A gain-of-function variant in dopamine D2 receptor and progressive chorea and dystonia phenotype**

Marlous C.M. van der Weijden MD^1,2,‡^, Dayana Rodriguez-Contreras PhD^3,‡^, Cathérine C.S. Delnooz MD, PhD^4^, Brooks G. Robinson PhD^5^, Alec F. Condon BA^5^, Michelle L. Kielhold BS^3^, Gilles N. Stormezand MD^6^, Kai Yu Ma Msc^1^, Claudia Dufke PhD^7^, John T. Williams PhD^5^, Kim A. Neve PhD^3,8^, Marina A.J. Tijssen MD, PhD^2,9,#,*^, Dineke S. Verbeek PhD^1,2,#^

^1^ Department of Genetics, University Medical Center Groningen, Groningen, The Netherlands

^2^ Expertise Center Movement Disorders Groningen, University Medical Center Groningen, Groningen, The Netherlands

^3^ Department of Behavioral Neuroscience, Oregon Health & Science University, Portland, Oregon, United States of America

^4^ Department of Neurology, Máxima Medical Center, Veldhoven, The Netherlands

^5^ Vollum Institute, Oregon Health & Science University, Portland, Oregon, United States of America

^6^ Department of Nuclear Medicine and Molecular Imaging, University Medical Center Groningen, The Netherlands

^7^ Institute of Medical Genetics and Applied Genomics, University Hospital Tuebingen, Tuebingen, Germany

^8^ Research Service, VA Portland Health Care System, Portland, Oregon, United States of America

^9^ Department of Neurology, University of Groningen, University Medical Center Groningen, Groningen, The Netherlands

^‡^ Shared first authors.

# Shared last authors.

*Corresponding author: m.a.j.de.koning-tijssen@umcg.nl

**Supplemental Data**

Online Supplemental Data include nine Tables, one Figure, and Supplemental Materials and Methods

**Supplemental Table 1. List of genes in dystonia gene panel**

*ADAR, ADCY5, ALDH5A1, ANO3, ARX, ATP13A2, ATP1A3, ATP7B, BCS1L, C10ORF2, C19ORF12, CACNA1B, CDKL5, CIZ1, COX10, COX15, COX20, CP, DDC, DLAT, DLD, FA2H, FBXO7, FOLR1, FOXG1, FTL, FUS, GCDH, GCH, GNAL, LRPPRC, MECP2, MTTP, NDUFA10, NDUFA12, NDUFA2, NDUFA9, NDUFAF2, NFUFAF5, NFUFAF6, NDUFS3, NDUFS4, NDUFS7, NDUFS8, NKX2-1, NPC1, NPC2, NUP62, PAH, PANK2, PARK2, PARK7, PCBD1, PDHA1, PDHB, PDHX, PINK1, PLA2G6, PLP1, PNKD, POLG, PRKRA, PRRT2, PTS, QDPR, RNASEH2A, RNASEH2B, RNASEH2C, SAMHD1, SCO2, SERAC1, SGCE, SLC16A2, SLC19A3, SLC20A1, SLC2A1, SLC30A10, SLC6A19, SLC6A3, SPG11, SPG7, SPR, SUCLA2, SUCLG1, SURF1, TACO1, TAF1, TH, THAP1, TIMM8A, TOR1A, TREX1, TUBB4A, VPS13A, WDR45*

*List of genes tested in the dystonia gene panel. Please note that the genes listed here were present in the 2017 dystonia gene panel (Department of Genetics of the UMCG, the Netherlands), In between testing of this panel and submission of this work, additional dystonia genes and chorea genes were identified, these genes were rechecked manually in retrospect and no mutations were identified.*

**Supplemental Table 2. Results repeat screening of *HTT*, HDL genes and benign hereditary chorea**

| Gene | Repeat Length or mutation found | Conclusion |
| --- | --- | --- |
| *HTT* | 18 and 20 repeats | Normal range |
| *ATN1* | 12 and 16 repeats | Normal range |
| *JPH3* | 13 and 15 repeats | Normal range |
| *NKX2-1* | No pathogenic mutation | No pathogenic mutation |
| *SCA17* | <43 repeats | Normal range |

*Repeat lengths or mutations in Huntington and Huntington like genes and benign hereditary chorea.*

**Supplemental Table 3. Primers used for screening *DRD2* gene**

| Exon | Forward sequence | Reverse sequence |
| --- | --- | --- |
| Exon 2 | 5’TGCAAGAGGCCCTCTCACTG 3’ | 5’GCTGGAGAAAGTGCTGGAGC 3’ |
| Exon 3 | 5’ACAAGACTTGCAGCTGCCTC 3’ | 5’CTGCACAGCATCACAGACACG 3’ |
| Exon 4 | 5' TCAGGCAAGCTTCATAGAGG 3' | 5' CATATCTGTGCCAGGGACTC 3' |
| Exon 5 | 5' TGTGGAATTATGGCCGGTGG 3' | 5' TTGCTGAGGTTTCCCAAGCC 3' |
| Exon 6 | 5' GATCTCTGAGCCCTTCTGCC 3' | 5' CACTTCATGCCTGCTTGGAG 3' |
| Exon 7 | 5' TGCCTCAGTGACATCCTTGC 3' | 5' GTGCCTGAGGAAATGCTAGC 3' |
| Exon 8 | 5' CACCGTCTTGGCATACGAGC 3' | 5' GCATGGAGCCAAGCGAACAC 3' |

**Supplemental Table 4. Shared variants between the two affected cases and absent in control predicted deleterious by three programs**

| **Chromosome** | **Position** | **Reference allele** | **Alternative allele** | **Genotype** | **Gene Symbol** | **cDNA** | **Protein** | **SIFT score** | **Polyphen2**  **score** | **Mutation Taster Prediction** | **gnomADv2.1.1** | **gnomADv3.0** | **dbSNP number** |
| --- | --- | --- | --- | --- | --- | --- | --- | --- | --- | --- | --- | --- | --- |
| 11 | 113286232 | T | A | T/A | *DRD2* | c.634A>T | p.I212F | 0 | 0.998 | Disease causing | Absent | Absent |  |
| 14 | 69341650 | C | A | C/A | *ACTN1* | c.2671G>T | p.G891C | 0 | 1 | Disease Causing | 60/249702 | 19/143286 | rs147023729 |
| 19 | 44039535 | G | C | G/C | *ZNF575* | c.434G>C | p.R145P | 0 | 1 | Disease causing | p.Arg145His;3/198798 | p.Arg145Cys;1/142998 |  |
| 20 | 62839672 | C | T | C/T | *MYT1* | c.1123C>T | p.R475W | 0 | 1 | Disease causing | 271/282404 | 154/143298 | rs144747841 |

*SIFT weight 0.0 to 0.05 damaging, PPH2 HumDivScore 0.85 to 1.0 deleterious.*

*Note: The variant in ZNF575 was a false positive Whole Exome Sequencing finding.*

**Supplemental Table 5. *In silico* prediction models for variant c.634A>T p.Ile212Phe in *DRD2***

| ***In Silico* prediction model** | **Score** | **Translation of the score into results** |
| --- | --- | --- |
| SIFT score | 0.00 | Affect protein function |
| Polyphen2 | 0.998 | Probably damaging |
| CADD | 32 | Disease-causing |
| MutationTaster | / | Disease-causing |
| pLI score | 0.75 | Mildly intolerant for loss-of-function and missense variants |
| Z-score | 2.62 | Mildly intolerant for loss-of-function and missense variants |

**Supplemental Table 6. Variants identified in *DRD2* within HD-like cohort using Sanger Sequencing.**

| **Genetic variant** | **Protein level long isoform** | **Exon (D2_L_)** | **Annotation** | **GnomAD** | **Presence in cohort analysis** | **Polyphen** | **SIFT** |
| --- | --- | --- | --- | --- | --- | --- | --- |
| c.50580G>A | p.Arg20Arg | Exon 2 | Synonymous | rs4986923 | Once | / | / |
| c.59762_59763 het_insC | Intronic | Between Exons 4-5 | Heterozygous Insertion | Not present | Once | / | / |
| c.59589G>C | p.Pro187Pro | Exon 5 | Synonymous | rs775101965 | Once | / | / |
| c.62410C>G | p.Ser311Cys | Exon 7 | Missense | rs1801028 | Four times | Probably damaging | Deleterious |
| c.62457A>G | p.Lys327Glu | Exon 7 | Missense | rs71653614 | Once | Benign | Tolerated |
| c.62517C>T | p.Pro347Ser | Exon 7 | Missense | rs200340299 | Once | Benign | Tolerated |

**Supplemental Table 7. Binding Potential values PET-scans**

| Individual | Age (years) | Binding potential  Caudate Nucleus | | Binding potential  Putamen | |
| --- | --- | --- | --- | --- | --- |
|  |  | Left | Right | Left | Right |
| Controls (N=10) | 30.6 ± 10.4 | 2.51 ± 0.50 | | 3.49 ± 0.43 | |
| III:8 | 60 | 1.4 | 1.4 | 1.9 | 1.9 |
| IV:4 | 30 | 2.0 | 2.1 | 2.8 | 2.6 |
| IV:5 | 29 | 1.8 | 1.8 | 2.8 | 2.7 |

*Binding potential (BP) from the [^11^C]raclopride-PET scan of the Caudate Nucleus and Putamen of three affected individuals and healthy controls. Individual III:8 showed reduced BP values compared to individuals IV:4 and IV:5. However, all BP values were considered to be within normal ranges when age-dependent decline was taken into account.*^1^

**Supplemental Table 8. Functional characterization of D2-I^212^F in HEK293 cells**

| **Receptor** | **G Protein Activation**  **(N=4)** | | **FSK-induced cAMP**  **(N=4-6)** | | **Arrestin Recruitment**  **(N=3-4)** | |
| --- | --- | --- | --- | --- | --- | --- |
|  | LogEC_50_ | E_max_  (% of WT) | LogIC_50_ | E_max_  (% of WT) | LogEC_50_ | E_max_  (% of WT) |
| D2_S_-WT | -7.58 ± 0.05 | 100 ± 3 | -8.70 ± 0.03 | 100 ± 1 | -7.90 ± 0.01 | 100 ± 3 |
| D2_S_-I^212^F | -8.42 ± 0.02^***^ | 99 ± 1 | -9.52 ± 0.03^***^ | 109 ± 5 | -8.33 ± 0.04^***^ | 68 ± 1^**^ |
| D2_S_ WT/I^212^F | -8.26 ± 0.04^***,†^ | 106 ± 1^††^ | -9.20 ± 0.02^***,†††^ | 98 ± 2 | -8.06 ± 0.01^***,†††^ | 87 ± 2^*,†††^ |
| D2_L_-WT | -7.62 ± 0.06 | 100 ± 4 | -8.48 ± 0.06 | 100 ± 8 | -7.84 ± 0.04 | 100 ± 3 |
| D2_L_-I^212^F | -8.32 ± 0.05^***^ | 95 ± 3 | -9.08 ± 0.06^***^ | 102 ± 8 | -8.32 ± 0.03^***^ | 48 ± 2^***^ |
| D2_L_-WT/I^212^F | -8.21 ± 0.06^**^ | 93 ± 0.5 | -8.75 ± 0.04^**,†††^ | 98 ± 7 | -8.01 ± 0.04^*,††^ | 81 ± 4^*,††^ |

*Quinpirole potency shown as logEC_50_ for G protein-activation and arrestin recruitment and as logIC_50_ for inhibition of forskolin (FSK) stimulated cAMP accumulation. E_max_ was calculated by subtracting basal response from maximal response at 10 min (G protein–activation and cAMP accumulation) and 20 min (arrestin recruitment) after adding the substrate coelenterazine h. E_max_ values are shown as percentage of the respective D2-WT. N, number of independent experiments performed for each BRET assay. Data presented as mean ± SEM.*

**, p<0.05; **, p<0.01; ***, p<0.001 compared to D2-WT. ^†^, p<0.05; ^††^, p<0.01; ^†††^, p<0.001 compared to D2-I^212^F.*

**Supplemental Table 9. D2 receptor density in HEK293 cells**

| **Receptor** | **G Protein Activation** | | **FSK-induced cAMP** | | **Arrestin Recruitment** | |
| --- | --- | --- | --- | --- | --- | --- |
|  | B_max_  (pmol/mg) | N | B_max_  (pmol/mg) | N | B_max_  (pmol/mg) | N |
| D2_S_-WT | 3.2 ± 0.6 | 10 | 4.1 ± 2.5 | 2 | 3.1 ± 0.2 | 8 |
| D2_S_-I^212^F | 1.1 ± 0.2*** | 8 | 1.7 ± 1.0 | 2 | 1.5 ± 0.1*** | 8 |
| D2_S_-WT/I^212^F | 2.6 ± 0.6 | 8 | 3.1 ± 2.2 | 2 | 2.5 ± 0.2** | 8 |
| D2_L_-WT | 2.0 ± 0.1 | 5 | 3.8 ± 0.8 | 3 | 2.6 ± 0.3 | 6 |
| D2_L_-I^212^F | 0.8 ± 0.1*** | 5 | 1.6 ± 0.1* | 3 | 1.0 ± 0.1*** | 6 |
| D2_L_-WT/I^212^F | 1.4 ± 0.1** | 5 | 3.0 ± 0.3 | 3 | 1.7 ± 0.3** | 6 |

*For most experiments included in Supplemental Table 8, replicate plates were prepared for analysis of receptor density. B_max_ values (Mean ± S.E.M., pmol/mg of membrane protein) were determined by saturation analysis of the binding of [^3^H]spiperone to a crude membrane fraction. In some BRET experiments, the number of cells was not sufficient to start replicate plates for binding. In some experiments, the number of replications (N) for radioligand binding was greater than the number of replications of the G protein activation and arrestin recruitment assays (Supplemental Table 8) because results from replicate plates of both BRET donor-only and BRET donor+acceptor conditions were included as separate measurements.*

**, p<0.05; **, p<0.01; ***, p<0.001 compared to D2-WT.*

**
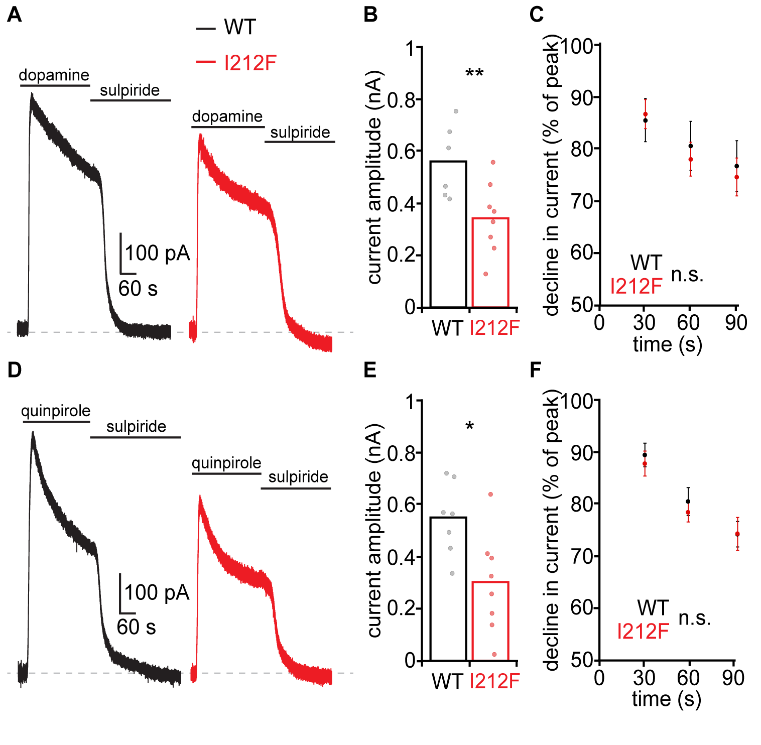
**

**Supplemental Figure 1. GIRK currents elicited by D2_S_ receptor activation in dopamine neurons. (A**) Example traces of D2_S_-WT (black) and D2_S_-I^212^F (red) receptor GIRK currents during the application of a saturating concentration of DA (100 µM). **(B)** Maximum current amplitudes elicited by dopamine (DA) were significantly larger in DA neurons expressing D2_S_-WT compared to D2_S_-I^212^F (N = 7 cells from 5 animals in D2_S_-WT, N = 8 cells from 7 animals in D2_S_-I^212^F; Student’s t-test; t = 3.1767, p = 0.007). **(C)** Desensitization (measured by decline in peak amplitude during continued agonist application) did not differ between neurons expressing D2_S_-WT versus D2_S_-I^212^F (N = 6 cells from 5 animals in D2_S_-WT, N = 8 cells from 7 animals in D2_S_-I^212^F. F; two way repeated measures ANOVA, p>0.05). **(D)** Example traces of D2_S_-WT (black) and D2_S_-I^212^F (red) receptor GIRK currents during the application of 10 µM quinpirole. **(E)** In neurons expressing D2_S_-WT, maximum currents elicited by the bath application of quinpirole (10 µM) were significantly larger than those in neurons expressing D2_S_-I^212^F (N = 7 cells from 6 animals in D2_S_-WT, N = 8 cells from 6 animals in D2_S_-I^212^F; Student’s t-tests; t = 2.8466, p = 0.014). **(F)** Using quinpirole as the agonist, the rate of desensitization was not significantly different between neurons expressing D2_S_-WT as compared to D2_S_-I^212^F (N = 7 cells from 6 animals in D2_S_-WT, N = 7 cells from 6 animals in D2_S_-I^212^F. F; two way repeated measures ANOVA, p>0.05).

*, p<0.05; **, p<0.01 compared to D2-WT. n.s., not significant.

**Online Supplemental Materials and Methods**

**Recombinant cDNA constructs**

cDNA encoding the human D2 receptor short isoform with a signal peptide and a Flag epitope at the receptor N-terminus (SF-hD2_S_)^2^ and the corresponding long isoform, SF-hD2_L_, were cloned into pcDNA3 and used to create SF-hD2_L_(I^212^F) and SF-hD2_S_(I^212^F) by site-directed mutagenesis as described below. Plasmids for measuring G protein activation (Gα_i1_-91-RLuc8, V1-Gß_1_ and V2-Gγ_2_) and inhibition of cAMP accumulation (pcDNA3L-His-CAMYEL; ATCC MBA-277) were described previously.^3,4^ For BRET assays to measure recruitment of arrestin3, plasmids containing the SF-hD2_L_ fused to an optimized version of *Renilla* Luciferase, RLuc8, human arrestin3 fused to mVenus, and human G protein-coupled receptor kinase-2 (hGRK2), were previously described.^3^ The I^212^F mutation was introduced in pcDNA3[SF-hD2_L/S_] and pcDNA3.1[SF-hD2_L_-RLuc8] using the QuikChange® II XL site-directed mutagenesis kit (Agilent Technologies, Santa Clara, CA, USA) with the following primers: Forward 5’- tgctggtctacatcaagttctacattgtcctccgc-3’; Reverse 5’-gcggaggacaatgtagaacttgatgtagaccagca-3’. To generate plasmids containing wild type and mutated D2_S_ isoforms fused to RLuc8 (pcDNA3.1[SF-hD2_S_-RLuc8] and pcDNA3.1[SF-hD2_S_(I^212^F)-RLuc8], respectively), pcDNA3.1[SF-hD2_L_-RLuc8] was digested with BamHI and PmlI (New England BioLabs, Ipswich, MA, USA) and the purified 6.5 kb fragment was ligated to each 1,145 bp insert generated by BamHI/PmlI digestion of pcDNA3[SF-hD2_S_] and pcDNA3[SF-hD2_S_(I^212^F)] using T4 DNA Ligase (New England BioLabs). For animal studies, a plasmid to generate Cre recombinase–dependent double-inverted open reading frame (DIO) adeno-associated virus (AAV) encoding hD2_S_ tagged at its C-terminus with a self-cleaving 2A peptide and EGFP was obtained from Jonathan Javitch (Columbia University, USA). This plasmid was modified by site-directed mutagenesis to introduce the I^212^F mutation using the QuikChange® II XL site-directed mutagenesis kit (Agilent Technologies, Santa Clara, CA, USA) with primers described above. Recombinant AAV8.2 vectors were produced by Virovek, Inc (Hayward, CA, USA). All new constructs were verified by DNA sequencing at the OHSU Vollum DNA Sequencing Core Facility (Portland, Oregon, USA).

**Cell culture and transfection conditions**

Human embryonic kidney 293 cells (HEK293), obtained from Caroline Enns (Oregon Health & Science University, USA), were maintained in Dulbecco’s modified Eagle’s medium (DMEM) supplemented with 10% fetal clone serum (FCS; Thermo Fisher Scientific, Waltham, MA, USA) at 37˚C in a 5% CO_2_ atmosphere. Eighteen hours before transfection, HEK293 cells were plated in 100-mm dishes at 60-80% confluence. Transient transfections were performed with polyethylenimine (PEI; MAX 40K reagent, Polysciences, Inc., Warrington, PA, USA) using a DNA:PEI ratio of 1:3 in Opti-MEM I (Gibco by Life Technologies, Logan, UT, USA). In most cases, two 100-mm petri dishes per condition were transfected to allow us to carry out BRET and radioligand binding assays using identically treated cells. Transfections were incubated for 5-6 h at 37˚C in the 5% CO_2_ humidified atmosphere, after which the medium was replaced by fresh DMEM plus 10% FCS. HEK293 cells were transfected with equal amounts of D2-WT or D2- I^212^F receptor DNA. Moreover, a group was included in which cells were co-transfected with a half-amount of each variant (WT and I^212^F) to mimic heterozygosity for the reference and rare alleles. This “heterozygous” treatment group is referred to as D2-WT/I^212^F.

To measure arrestin3 recruitment, HEK293 cells were co-transfected with plasmids contain hGRK2 (2 µg), mVenus-Arr3 (2.5 µg), and the WT or I^212^F-mutated D2 receptor tagged to RLuc8 (0.25 µg). For G protein activation, cells were co-transfected with WT or I^212^F-mutated D2 receptor (0.5 µg), and the G protein subunits Gα_i1_-91-RLuc8 (0.2 µg), V1-Gß_1_ (2 µg) and V2-Gγ_2_ (2 µg). Cells were harvested for BRET studies (see main text) and radioligand binding assays 48 h post-transfection.

D2-mediated inhibition of cAMP was measured using a BRET-based cAMP sensor, CAMYEL.^4^ Cells transfected with the WT or I212F-mutated D2 receptor (0.5 µg) and CAMYEL (2.5 µg) were treated with several concentrations of quinpirole followed by addition of 10 µM forskolin and luciferase substrate. Quinpirole-mediated inhibition of forskolin-induced cAMP accumulation was measured after 10 min.

**D2 receptor radioligand binding**

Membrane expression of the receptors was evaluated essentially as previously described.^3^ Cells were lysed in ice-cold hypotonic buffer (1 mM HEPES, 2 mM EDTA, pH 7.4), scraped from the plate, and centrifuged at 17,000 × *g* at 4° C for 20 min. The resulting pellet was resuspended in Tris-buffered saline (TBS: 50 mM Tris, 120 mM NaCl, pH 7.4) and homogenized for 10 sec using a Polytron homogenizer (Brinkmann Instruments, Westbury, NY). Protein determination was performed using the BCA Protein Assay Kit (Thermo Scientific). Samples were incubated in TBS containing 0.002% BSA and [^3^H]spiperone at 37˚C for 1 h in a final volume of 1 ml before addition of ice-cold buffer and vacuum filtration. Nonspecific binding was assessed using (+)-butaclamol (2 µM).

**Mouse studies**

All studies were conducted in accordance with the Institutional Animal Care and Use Committees at the VA Portland Health Care System (VAPORHCS) and Oregon Health & Science University (OHSU). Thirty-one mice (16 male and 15 female, 60-90 days old) were used in this study. Auto-D2-KO mice were bred at the VAPORHCS Veterinary Medical Unit by crossing *Drd2^loxP/loxP^* mice,^5^ obtained from Jonathan Javitch (Columbia University, USA), with heterozygous B6.SJL-*Slc6a3^tm1.1(cre)Bkmn^*/J mice^6^ obtained from the Jackson Laboratory (JAX stock #006660). All mice were maintained on a C57BL/6 background. Mice were housed in standard plastic containers on a 12 hr light/dark cycle with food and water available *ad libitum*. For expression of recombinant D2_S_ receptors in dopamine neurons, auto-D2-KO mice were immobilized in a stereotaxic alignment system after i.p. injection of an anesthesia cocktail consisting of 7.1 mg/kg xylazine, 71.4 mg/kg ketamine, and 1.4 mg/kg acepromazine (10 ml/kg). Mice received bilateral 500 nl injections in the ventral tegmental area with Cre recombinase-dependent double-inverted open reading frame (DIO) adeno-associated virus (AAV) encoding hD2S variants tagged at its C-terminus with a self-cleaving 2A peptide and GFP. Injections were given at a rate of 200 nl/min, with the injection needle left in place for an additional 5 min before it was slowly withdrawn. The coordinates for injections were AP −3.26 mm, ML ±1.2 mm, DV −4.0 mm. After injections, mice recovered in individual (male) or group (female) housing for 2-3 weeks to allow for expression. Infected neurons were identified in the slice by visualization of green fluorescence.

**Electrophysiology in mouse midbrain slices**

Whole-cell voltage clamp recordings (holding potential −60 mV) were made as previously described.^7^ Experiments were conducted blinded to D2S receptor genotype. Mice were deeply anesthetized with isoflurane and killed by decapitation. Brains were removed quickly and placed in warmed (~34°C) physiologically equivalent saline solution (modified Krebs buffer) containing (in mM): 126 NaCl, 2.5 KCl, 1.2 MgCl2, 2.4 CaCl2, 1.4 NaH2PO4, 25 NaHCO3, and 11 D-glucose with MK-801 (10 μM), and cut horizontally (220 μm) using a vibrating microtome (Leica Microsystems, Buffalo Grove, IL, USA). Slices recovered at 30°C in vials with 95/5% O2/CO2 saline with MK-801 (10 μM) for at least 30 min prior to recording. Slices were then mounted in a recording chamber and perfused at a rate of ∼3.0 ml/min with 35–36°C modified Krebs buffer. Recordings were made exclusively from neurons in the substantia nigra, pars compacta, identified visually by their location lateral to the medial terminal nucleus of the accessory optic. Dopamine neurons were identified by location and electrophysiological properties, namely the presence of spontaneous pacemaker firing of broad (∼2 ms) action potentials at 1-5 Hz in cell-attached mode,^8^ and characteristic passive membrane properties including capacitance and resting conductance.^9^ Neurons were also confirmed to be virally expressing D2 receptors through the visualization of EGFP encoded by the virus. Recordings were obtained with large glass electrodes with a resistance of 1.3-1.9 MΩ when filled with internal solution containing (in mM): 115 K-methanesulfonate, 20 NaCl, 1.5 MgCl_2_, 10 HEPES (K), 2 ATP, 0.2 GTP, 10 phosphocreatine, and 10 BAPTA (K4); pH 7.33-7.40, 275-288 mOsm. Within 2 min of break-in, membrane capacitance, series resistance, and input resistance were measured with the application of 3 pulses (±2 mV for 50 ms) averaged before computation using the Axograph (sampled at 50 kHz, filtered at 10 kHz). Series resistance was monitored to ensure sufficient and stable electrical access to the inside of the cell throughout the experiment (<12 MΩ). Cells were dialyzed with internal solution for >10 min prior to drug application. Drugs were applied through bath perfusion. Data were acquired using AxoGraph software (AxoGraphX, Berkeley, CA, USA) and Chart 7 (AD Instruments, Colorado Springs, CO, USA) and were post hoc filtered.

For stimulated inhibitory post-synaptic currents (IPSCs), a monopolar glass electrode was inserted into the slice near the neuron being recorded. Using a stimulus isolator (World Precision Instruments, Sarasota, FL, USA), electrical stimulations were applied ranging from 10 to 100 µA. Stimuli were 0.2 ms in length and applied either individually or in trains of 5 pulses at 40 Hz. To capture and analyze spontaneous IPSCs, the Event Detection program in Axograph software was used, as previously described.^2^ In short, 60 episodes were recorded with Axograph at 10 kHz. Episodes were then filtered at 1 kHz and decimated so that each point represented an average of 10. Template IPSCs for D2_S_-WT and D2_S_-I212F were created by averaging obvious spontaneous events. Using a sliding window method, these templates were scanned through the episodes. To be detected, events had to be greater than 2.1x the standard deviation of the noise in the recording. Detected events were manually examined for quality assurance.

**References**

1. Nakajima S, Caravaggio F, Boileau I, et al. Lack of age-dependent decrease in dopamine D 3 receptor availability: A [11C]-(+)-PHNO and [11C]-raclopride positron emission tomography study. *J Cereb Blood Flow Metab.* 2015; 35(11):1812-8

2. Gantz SC, Bunzow JR, Williams JT. Spontaneous inhibitory synaptic currents mediated by a g protein-coupled receptor. *Neuron.* 2013;78(5):807-12

3. Clayton CC, Donthamsetti P, Lambert NA, Javitch JA, Neve KA. Mutation of three residues in the third intracellular loop of the dopamine D2 receptor creates an internalization-defective receptor. *J Biol Chem.* 2014; 28;289(48):33663-75

4. Jiang LI, Collins J, Davis R, Lin KM, et al. Use of a cAMP BRET sensor to characterize a novel regulation of cAMP by the sphingosine 1-phosphate/G13 pathway. *J Biol Chem*. 2007; 6;282(14):10576-84

5. Bello EP, Mateo Y, Gelman DM, et al. Cocaine supersensitivity and enhanced motivation for reward in mice lacking dopamine D2 autoreceptors. *Nat Neurosci.* 2011; 10;14(8):1033-8

6. Bäckman CM, Malik N, Zhang YJ, et al. Characterization of a mouse strain expressing Cre recombinase from the 3′ untranslated region of the dopamine transporter locus. *Genesis*. 2006; 44(8):383-90

7. Gantz SC, Robinson BG, Buck DC, et al. Distinct regulation of dopamine D2S and D2L autoreceptor signaling by calcium. Elife. 2015; 26;4:e09358

8. Ford CP, Mark GP, Williams JT. Properties and opioid inhibition of mesolimbic dopamine neurons vary according to target location. *J Neurosci*. 2006; 8;26(10):2788-97

9. Gantz SC, Ford CP, Neve KA, Williams JT. Loss of Mecp2 in substantia nigra dopamine neurons compromises the nigrostriatal pathway. *J Neurosci*. 2011; 31;31(35):12629-37
